# Cognitive and Interpersonal Factors Affecting Social Adjustment of University Students in Pakistan

**DOI:** 10.3390/ijerph20010655

**Published:** 2022-12-30

**Authors:** Saima Kayani, Niaz Muhammad Aajiz, Khisro Kaleem Raza, Sumaira Kayani, Michele Biasutti

**Affiliations:** 1 Faculty of Arts, Social Science and Education, Sarhad Universit of Science and Information Technology, Peshwar 25000, Pakistan; 2 Department of Psychology, College of Education, Zhejiang Normal University, Jinhua 321004, China; 3 Department of Philosophy, Sociology, Education and Applied Psychology (FISPPA), University of Padova, 35139 Padova, Italy

**Keywords:** social adjustment, self-efficacy, teachers’ support and peer support

## Abstract

Cognitive and interpersonal factors play an important role in the social adjustment of students. Factors affecting the social adjustment of university students have been verified in different cultures. However, no study has tested a concurrent model with the study variables in the Pakistani context. This study aimed to investigate the effect of personal and interpersonal factors on the social adjustment of university students in Pakistan. Three hundred participants from the Azad Jammu and Kashmir regions of Pakistan responded on a questionnaire package containing self-reported measures on social self-efficacy, social anxiety, teachers’ social support, and peers’ social support. The results indicate that self-efficacy, teachers’ support, and peer support have a significant positive effect on the social adjustment of university students, suggesting that an enhanced self-efficacy, and increased teachers’ and peers’ support would increase social adjustment. However, academic anxiety is inversely associated with social adjustment, suggesting that a higher anxiety level would result in a reduction in social adjustment. Students should be given more opportunities to enhance self-efficacy, obtain social support, and reduce social anxiety.

## 1. Introduction

### 1.1. Social Adjustment

Social adjustment is the extent to which a person engages in a proper social activity and adjusts to his or her current social surroundings [1]. The students have to develop the ability for adaptation, which allows them to mature into responsible members of society. In psychology, social adjustment is the skill to adapt to changes in the physical, vocational, and social environment. In other words, according to society, social adjustment is the behavioral process of reconciling competing demands or requirements challenged by environmental impediments [2]. Important aspects of social adjustment include integrating into the social life of a university, a college, a city, and a nation, establishing an environmental network, and regulating social freedoms in a particular context. Social adjustment is crucial for everyone, but especially for undergraduates in the university environment [3].

### 1.2. Social Adjustment of University Students and the Related Factors

Social adjustment is a persistent obstacle for students entering into a new environment [4]. The worldwide rate of psychological indisposition among university students is rather high [5]. However, male students are better socially adjusted and possess more college affiliation-related interpersonal support than their female counterparts [6,7]. During the transition period from college to university, students may face many challenges, including new environment, teachers, friends, lifestyle, and changed academic setup [8]. For instance, research stated that students in their new environment confront a range of social and academic transition challenges [9]. Common issues include difficulties with communication, accommodation-related stress, organizational culture, and friendships. If university students are unable to adequately manage these new problems, they become more susceptible to psychosocial issues [10]. To combat these issues, it is imperative to understand factors affecting the social adjustment of university students. Social, cognitive, and environmental factors impact a person in society, according to Bandura’s social cognitive theory [11]. Research also authenticates the interaction of personal and interpersonal factors contributing to the adaptation to the university environment [12]. Therefore, the most important factors influencing social adjustment can be cognitive- and interpersonal-level variables.

### 1.3. Cognitive Factors

Cognitive or personal factors influencing the social adjustment of university students are social self-efficacy and social anxiety. It is believed that social self-efficacy would help in social adjustment in a positive way. For instance, Ahmed and his coworkers [5] found a significant direct association between social self-efficacy and social adjustment of university students in Pakistan. Other empirical research also reported that social adjustment is increased with an enhanced social self-concept in different cultures [12,13,14]. Furthermore, the students’ mental health is reported to have a positive association with their social adjustment at a university in Thailand [15]. Students with mental health issues such as anxiety disorders usually develop a feeling of isolation and poor mental health. For example, a multilevel analysis resulted in a negative effect of social anxiety on social adjustment among a sample of 668 students from Portugal, indicating an increase in social withdrawal with increased social anxiety [16]. The research also reported that girls were more anxious than boys. Research also reported that high level of social anxiety impede social adjustment among university students with girls exhibiting more anxiety than boys [17,18]. Hence, it was expected that self-efficacy and social anxiety would also play a role in Pakistan where the social adjustment problems of university students are higher than in other societies [19].

### 1.4. Interpersonal Factors

Interpersonal variables affecting the social adjustment of university students are teachers’ social support and friends/peers’ social support. According to Ahmed and others [20], social support may be described in terms of participation in social activities and satisfaction with the university’s social environment. A research from Korea investigated factors affecting the academic and social adjustment of university students and found interpersonal support as a significant contributor to social adjustment [21]. Birzina and his colleagues [12] reported that interpersonal relationships have an impact on the students’ ability to adjust with the other students, leading to overall adjustment at university. For example, perceived attachment to parents and friends was proven to be a major contributor to an effective transition among young adults enrolling in universities in previous studies [22]. It is believed that teachers provide cognitive and emotional support to a student in any situation, maintain an optimistic mindset, emphasize the positivity of the surroundings, acknowledge the students’ emotions and their living experiences, integrate their interests and experiences in the new environment, and engage them in activities to resolve issues they may have developed during the school experience [23]. However, male students with a greater level of perceived interpersonal support tend to adapt better to varied social circumstances, while females have greater difficulty in developing social ties [24]. As a result, the adjustment of female university students was primarily influenced by their social interactions, interpersonal support, and social experiences at the university [24]. Furthermore, peer support is perceived as encouraging others to achieve long-term goals, providing emotional support, sharing information, educating skills, feeding practical assistance, and linking individuals with resources, opportunities, support networks, and other individuals [25]. For instance, Zhang and is coauthors [26] found the highest score on social adaptation with interpersonal support in a sample of Chinese students. [27] also found that peers have a significant impact on the social and emotional development of students at a university. Moreover, perceived attachment to parents and friends was proven to be a major contributor to an effective transition among young adults enrolling in universities in previous studies [22]. Conversely, a study from Zimbabwe showed a negative impact of friendship on the social adjustment of university students [28], indicating inconsistent findings from different societies. Therefore, it is hypothesized that interpersonal support may have a different effect on the social adjustment of university students in Pakistan.

### 1.5. Current Study

**Figure 1:**
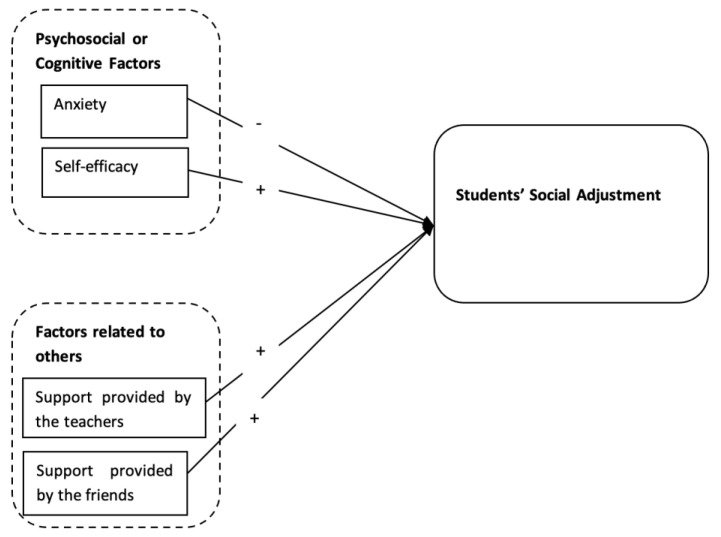
Theoretical framework.

Factors affecting the social adjustment of university students have long been tested in the past. However, the previous research has focused more on only the social adjustment patterns of university students in Pakistan [6]. A few researchers have investigated the relationship of social adjustment with self-efficacy [5] and self-concept among university students in Pakistan [13]. Another recent study has investigated the association between interpersonal support and social adjustment problems in university students [29]. However, one of the gaps in the current literature is considering the relationship among cognitive and interpersonal factors with social adjustment in a concurrent model in a Pakistani context where most of the students have psychological disorders such as anxiety and depression [19]. Furthermore, the literature on the social cognitive aspects of university students is scarce in the Azad Jammu and Kashmir regions of Pakistan. Hence, there is a dire need of conducting a study with these factors. Therefore, based on the social cognitive theory [11], the current research is planned to investigate the impact of cognitive and interpersonal factors on university students’ social adjustment as reported in Figure 1. The following hypotheses have been formulated for the current study:

**Hypothesis** **1 (H1).** *Cognitive factors such as social self-efficacy would significantly increase social adjustment of university students.*

**Hypothesis** **2 (H2).** *Cognitive factors such as social anxiety would significantly decrease social adjustment of university students.*

**Hypothesis** **3 (H3).** *Interpersonal factors such as teachers’ support would positively affect social adjustment of university students.*

**Hypothesis** **4 (H4).** *Interpersonal factors such as peers’ support would positively affect social adjustment of university students.*

## 2. Materials and Methods

Based on the social cognitive theory, this study is planned to investigate social cognitive factors affecting social adjustment of university students. The major aim is to see the effect of cognitive and interpersonal factors on social adjustment. A cross-sectional design is chosen for the current study. The following section contains the materials and methods for the study.

### 2.1. Participants

**Figure 2:**
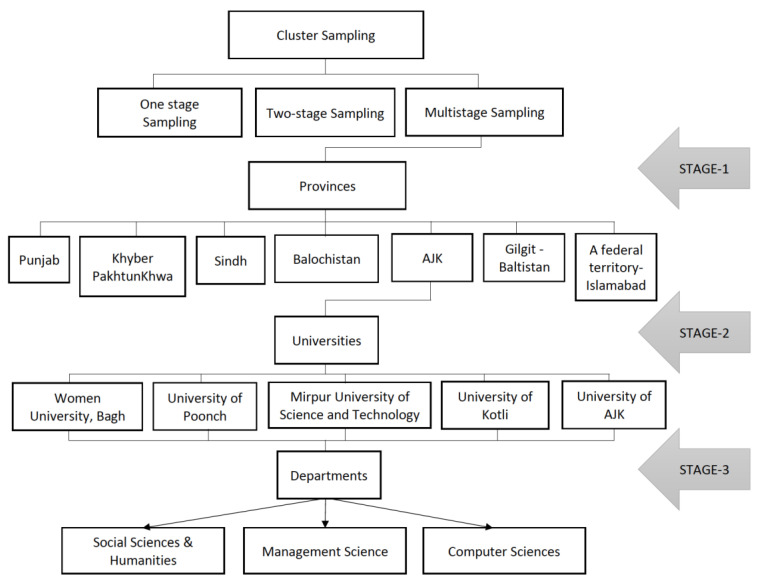
Sampling technique.

The population of this study was university students. The study’s participants were from public sector universities of Azad Jammu and Kashmir (AJK), Pakistan. The first-year university students were our target population. The selection of first-year students was based on the fact that this is the pre-university stage of students’ lives, and they face the majority of social adjustment issues at this time. The total number of undergraduate students was 975, all of whom were enrolled in the selected departments of these universities. The current study employed the multistage cluster sampling technique (see Figure 2). At the first stage, our clusters were the 6 provinces of Pakistan and one federal territory out of which only the AJK province was selected. At the second stage, there were all universities from AJK. Then, the departments from these universities were chosen at the third stage. Finally, the questionnaires were distributed to the students of the selected departments based on an informed consent. It is believed that the sample size of 278 is enough if the population is 1000 [30]. Hence, the researcher distributed 330 questionnaires to students from the designated representative sample of 1st-year students (slightly greater than the minimum number to account for non-response). However, 300 questionnaires were returned, resulting in a response rate of 91%.

Table 1 Presents the characteristics of participants in the study.

### 2.2. Questionnaires

#### 2.2.1. Social Adjustment

A total of 27 questions were created to assess university students’ social adjustment. Expert examinations and statistical measurements led to the exclusion of thirteen items. Finally, a tool was developed with 14 items scored on a 5-point Likert scale (1 = Strongly Agree to 5 = Strongly Disagree). “I am generally engaged in university social activities,” “I am content with my degree of social participation,” and “I can easily interact with others at university,” are some examples.

#### 2.2.2. Anxiety

For testing university students’ social interaction anxiety, a pool of 41 items was created. Following the experts’ examination, sixteen items were eliminated. A total of 13 items were put onto a single factor, while the rest were not. “I avoid talking to strangers,” “I feel peaceful when I readily engage with others,” and “I feel anxious in social situations” are some of the examples. All items are assessed on a 5-point Likert scale, with 1 indicating strong agreement and 5 indicating strong disagreement.

#### 2.2.3. Self-Efficacy

The social self-efficacy of university students was assessed using a set of 34 items. Following the experts’ examination, seven elements were eliminated. A single factor was loaded on ten things, whereas others were not. “I can meet people and make friends,” and “I can handle social interaction quite well,” are two examples. All items are assessed on a 5-point Likert scale, with 1 indicating strong agreement and 5 indicating strong disagreement.

#### 2.2.4. Teachers’ Support

At the university level, a pool of 17 questions was created to assess teachers’ support for social adjustment. Following the experts’ examination, five elements were eliminated. Three elements were loaded on a single factor, but the other nine were not. The tool did not include any items that were not loaded on any factor. Finally, a tool with nine components was created for instructors and peers to use. “I feel like my professors would be empathetic if I had a problem,” and “The teachers’ inquiries help me to comprehend,” are two examples. All items are graded on a 5-point Likert scale, with 1 indicating strong agreement and 5 indicating strong disagreement.

#### 2.2.5. Peers’ Support

For assessing peers’ assistance for adjustment in the university’s social environment, a pool of 21 items was created. Following the experts’ review, 9 elements were eliminated. On a single factor, ten elements were loaded, whereas two were not. “I have built individual links with the many peers in class.” and “I feel like I am a part of a helpful professional network.” are two examples. All items are assessed on a 5-point Likert scale, with 1 indicating strong agreement and 5 indicating strong disagreement.

### 2.3. Data Analysis Techniques and Procedure

The researcher personally visited the sample institutions for data collection to gather data for the study. A questionnaires package containing scales on self-efficacy, social anxiety, teachers’ support, and peers’ support was provided to the participants. The participants were asked to sign an informed consent before attempting the survey. After that, data were analyzed by using SPSS ver. 21. Data were screened for missing values and outliers before analysis. First, validation process of all the tools was executed. Exploratory factors analysis was used to explore factor structures of the tools. After that, gender difference analysis was provided to see whether social adjustment patterns were different across different genders. Then, correlation among study variables was presented. At the end, regression analysis was applied to see the effect of cognitive and interpersonal factors on social adjustment of university students.

## 3. Results

### 3.1. Validation of Instruments

The data were verified for missing values and normal distribution. All the cases with missing values were deleted to screen data. Skewness and kurtosis show acceptable standard (+2) exhibiting normality of data [31]. Coefficient of variability was also verified and the acceptable values were attained. Table 1 represents the validation of the instruments used in the study. Exploratory factor analysis (EFA) with direct oblimin was applied to extract factors of each instrument. Each instrument was found with single factor containing 14 items for SA (KMO = 0.84, *p* < 0.05, 47% variance explained), 11 items for SE (KMO = 0.75, *p* < 0.05, 39% variance explained), 9 items for TS (KMO = 0.890, *p* < 0.05, 48.94% variance explained), 10 items for PS (KMO = 0.785, *p* < 0.05, 38% variance explained), and 13 items for anxiety (KMO = 0.824, *p* < 0.05, 42% variance explained). The results for EFA were significant. However, the variance explained for SE and PS was below 40%, which may be one of the limitations of the study. The items which could not load on any factor were excluded. Factor loadings were used to assess the convergent validity and discriminant validity of the tools by using James Gaskin stats tool for validation [32]. Gaskin pointed out that convergent validity can be assessed through average variance extracted (AVE) and composite reliability (CR). If AVE > 0.5 and CR > AVE, the tool is considered to have convergent validity. Furthermore, discriminant validity is attained when the AVE is greater than the maximum shared variance (MSV) and √AVE > the inter-construct correlations [33]. Cronbach’s alpha and composite reliability shows the that all the study instruments were reliable. Hence, all the tools are valid and reliable as shown in Table 2.

### 3.2. Effect of Gender on Social Adjustment

In Table 3, *t*-test was applied for gender, and it was revealed that there was a significant difference between the social adjustment of male and female students. However, it is surprising that males (*M* = 41.33; *SD* = 10.39) have a lower social adjustment than females (*M* = 50.11; *SD* = 7.45), *t*(298) = 6.983, *d* = 0.97. The result from Cohen’s d shows that the social adjustment of females is significantly higher than that of males with a larger effect size [34]. Hence, gender was controlled for the study.

### 3.3. Correlation Analysis

A partial correlation was run to determine the relationship between the students’ social adjustment self-efficacy, teachers’ support, peers’ support, and social anxiety controlling for gender. The results exhibit that there was a moderate, negative partial correlation between social adjustment (48.71 ± 8.59) and social anxiety (35.12 ± 9.04) whilst controlling for gender (1.84 ± 0.37), which was statistically significant, *r*(297) = −0.362, *n* = 300, *p* < 0.001; there was a high, positive partial correlation between social adjustment (48.71 ± 8.59) and self-efficacy (38.26 ± 7.05) while controlling for gender (1.84 ± 0.37), which was statistically significant, *r*(297) = 0.601, *n* = 300, *p* < 0.001; there was a high, positive partial correlation between social adjustment (48.71 ± 8.59) and teachers’ support (35.03 ± 7.12) while controlling for gender (1.84 ± 0.37), which was statistically significant, *r*(297) = 0.524, *n* = 300, *p* < 0.001; there was a high, positive partial correlation between social adjustment (48.71 ± 8.59) and peers’ support (35.05 ± 6.82) while controlling for gender (1.84 ± 0.37), which was statistically significant, *r*(297) = 0.537, *n* = 300, *p* < 0.001. On the other hand, zero-order correlations showed that there was a statistically significant, moderate negative correlation between social adjustment and social anxiety (*r*(298) = −0.422, *n* = 300, *p* < 0.001), a statistically significant high positive correlation between social adjustment and self-efficacy (*r*(298) = 0.652, *n* = 300, *p* < 0.001), a statistically significant high positive correlation between social adjustment and teachers’ support (*r*(298) = 0.603, *n* = 300, *p* < 0.001), and a statistically significant high positive correlation between social adjustment and peers’ support (*r*(298) = 0.698, *n* = 300, *p* < 0.001). The mean value of gender (1.84 ± 0.37) suggests that the sample of participants was more on the female side rather than representing the population as a whole. This may be one of the limitations of the study.

### 3.4. Regression Analysis (Hypotheses Testing)

Table 4 describes the results of regression analysis and hypotheses testing. First, it was hypothesized that social self-efficacy would significantly increase the social adjustment of university students. It is indicated that social adjustment is significantly positively affected by self-efficacy (β = 0.796, t = 14.862, *p* < 0.05), hence confirming H1. Second, it was predicted that social anxiety would significantly decrease the social adjustment of university students. It is evident from the results that anxiety (β = −0.402, t = 8.047, *p* < 0.05) has a negative impact on the social adjustment of the students, hence supporting H2. Third, it was anticipated that teachers’ support would positively affect the social adjustment of university students. The results exhibited that teachers’ support positively affects social adjustment (β = 0.729, t = 13.057, *p* < 0.05), hence confirming H3. Fourth, it was hypothesized that peers’ support would positively affect the social adjustment of university students. The results show that peers’ support had a significant positive effect on social adjustment (β = 0.753, t = 12.864, *p* < 0.05). Hence, H4 is also supported.

## 4. Discussion

The current study aimed to investigate the effect of social self-efficacy, social anxiety, teachers’ and peers’ support on the social adjustment of university students. The study contributes to the social cognitive theory by authenticating the conceptual model of association of social and cognitive factors with social adjustment. The hypothetical model in our study suggests the importance of a theoretical framework predicting social adjustment in university students. Furthermore, the present research indicates a novelty in presenting a social cognitive model of social adjustment in the Pakistani context.

First, a validation process of all instruments was presented. The results from EFA and CFA indicated that all tools have significant validity and reliability. The only thing which matters was the less than 40% variance explained by self-efficacy and peers’ support. This may be one of the limitations of the study. After that, gender difference analysis was verified through *t*-test to see whether social adjustment patterns were different depending on sex. It was found that there was significant difference between boys and girls for social adjustment. However, the results are rather astonishing as girls exhibited a higher social adjustment than boys. This result contradicts previous findings which presented male students as showing more social adjustment than females [6,7]. The reason for this may be that the male students in Pakistan are more susceptible to psychological distress, specifically depression and anxiety [35], thus, possessing lower social adjustment. Another reason may be a smaller number of boys (*n* = 28) in the sample in comparison to girls (*n* = 252). It is suggested to conduct more research by taking larger samples in the future to authenticate the generalizability of the results.

Major findings revealed that self-efficacy significantly positively affected the social adjustment of students. This is consistent with the previous results according to which social self-efficacy was positively associated with the social adjustment of university students [5]. The results also authenticate the social cognitive model of social adaptation [36], uncovering social self-efficacy as determining better adjustment in an environment. The studies from other societies also reported that social adjustment is increased with an enhanced social self-concept in different cultures [12,13,14]. This implies that the ability to adjust in the social environment leads to positive change in social adjustment.

The second key finding of this research is that social anxiety decreases social adjustment in university students. This result echoes the previous study reporting that social anxiety is negatively associated with the social adjustment of students leading to social withdrawal and maladjustment, with boys possessing more social adjustment than girls [16,18]. The findings are also in line with the work of Khalid and his colleagues, [18] and Peleg [17] who found that a high level of social anxiety impedes social adjustment among university students, with girls exhibiting more anxiety than boys. This implies that students with less social anxiety would be better at social adjustment in a university.

Furthermore, we have identified that social support from teachers has a strong positive effect on the social adjustment of students. This finding is in accordance with the previous literature. For instance, a research from Korea investigated the factors affecting the academic and social adjustment of university students and found that teachers’ support significantly positively affect social adjustment [21]. Birzina and his colleagues [12] also reported consistent findings with the current research that teachers’ social support has a positive impact on students’ ability to adjust with the other students, leading to overall adjustment at university as teachers provide cognitive and emotional support to a student in any situation [23]. These results imply that the teachers’ social support for students at a university significantly increases their social adjustment.

In addition, peer support positively predicted the social adjustment of students in the present study. This finding is associated with the perspective of Orben and his colleagues [37], uncovering that peers profoundly impact others’ social and emotional development by playing the role of social models and reinforcing agents. These results also authenticate the previous empirical research with Chinese students exhibiting peer support positively affecting social adaptation [26]. This implies that interaction with peers helps students acquire the interpersonal skills essential for successful adjustment outside the family and seek independence.

## 5. Strengths and Limitations

This study has several strengths. For instance, this study has specifically evaluated personal and interpersonal factors affecting the social adjustment of university students in the context of Pakistan. This study has provided the evidence necessary to validate the social cognitive model of social adjustment in Pakistan, where it has not been tested before with the current variables. This study could specifically impact the university environment by taking social cognitive elements into consideration for the social learning of the students. This invites us to lay emphasis on enhancing self-concepts such as self-efficacy to reduce anxiety and increase social support for the social adjustment of the students at a university. Despite this, this study is cross-sectional in nature. Furthermore, this study was conducted in only one region of Pakistan, which may affect the generalizability of the results. Moreover, all the tools used in the current study were self-reported measures. Using objective measures may give different results. One important limitation of this study is the higher number of females than males, which may affect the generalizability of the results to the whole population. In addition, intervention and longitudinal studies in the future are required to better understand the effect of cognitive and social factors on the social adjustment of university students in Pakistan.

## 6. Research Implications

The findings of the current study imply that positive self-concepts such as self-efficacy, decreased social anxiety, and increased interpersonal support from teachers and peers enhance social adjustment among university students. These findings are in line with the social cognitive theory, indicating that social and cognitive factors positively contribute to social adaptation [36]. As far as we know, this would be the pioneer study in testing the social cognitive model of social adjustment in Pakistani university students. This research demonstrates that social adjustment is positively influenced by self-efficacy, teachers’ support, and peers’ support; while negatively affected by social anxiety. Thus, the current study provides evidence for the protective power of self-concept and interpersonal factors for social adjustment among university students in Pakistan.

This study also has practical implications. The research has called our attention to the importance of cognitive and interpersonal factors promoting the social adjustment of university students. The current study has identified that personal and social factors play valuable roles in enhancing the social adjustment of students. Therefore, it is suggested to reduce social anxiety, enhance self-efficacy, and increase social support by teachers and peers to promote social adjustment. During the period of transition from college to university, students may confront several obstacles, including a new environment, professors, friends, lifestyle, and academic structure. Hence, this is the responsibility of an educational institution to bring change at an individual’s personal and social level to make him or her socially adjusted in the university environment. It is also recommended that institutions consider teachers’ professional development in the domain of encouraging interpersonal support, self-efficacy, and reducing anxiety among students. Future research with more cognitive and interpersonal factors, and with mediation and moderation analysis, are suggested to add more to the body of knowledge. Furthermore, intervention studies aiming at the promotion of social adjustment through social cognitive factors could be taken into consideration.

## 7. Conclusions

The current research reports on the effect of cognitive and interpersonal factors on the social adjustment of university students in Pakistan. This research showcased the validation of the social cognitive model of social adaptation. This study’s research model was based on the evidence gathered from 300 students enrolled in social science, computer science, and business studies in Pakistani universities. After analyzing data in SPSS, we found that social anxiety negatively affected social adjustment, self-efficacy positively influenced social adjustment, and interpersonal support from teachers and peers positively predicted social adjustment. Therefore, our findings reveal that personal and interpersonal factors significantly contribute to the social adjustment of university students in the Pakistani context. This research suggested that students who have less social anxiety, increased self-efficacy, and more social support would be better at social adjustment.

## Figures and Tables

**Table 1:** Demographic characteristics.

Characteristic (*n* = 300)	Frequency	Percentage
**Gender**		
Male	48	16
Female	252	84
**Age**		
17–20	225	75
21–24	72	24
25–28	3	1
**Geographic area**		
Urban	164	54.7
Rural	136	45.3
**Social class**		
Lower	6	2
Middle	176	58.7
High	118	39.3
**Financial constancy**		
Very unstable	45	15
Moderately stable	192	64
Very stable	63	21
**University**		
University 1	91	30.3
University 2	61	20.3
University 3	55	18.3
University 4	57	19
University 5	36	12
**Department**		
Business administration	45	15.0
Computer science	53	17.7
Economics	46	15.3
Education	87	29.0
English	31	10.3
International relations	38	12.7
**Accommodation**		
Inside campus	30	10
Outside campus	270	90

**Table 2:** Validation of instruments.

Variables and Items	Items and Estimates														AVE	√AVE	C.R.	α
SA	SA1	SA2	SA3	SA4	SA5	SA6	SA7	SA8	SA9	SA10	SA11	SA12	SA13	SA14	0.53	0.72	0.94	0.79
Estimate	0.743	0.63	0.74	0.753	0.704	0.74	0.669	0.659	0.771	0.738	0.762	0.734	0.773	0.711				
SE	SE1	SE2	SE3	SE4	SE5	SE6	SE7	SE8	SE9	Se10	SE11				0.54	0.74	0.95	0.72
Estimate	0.824	0.685	0.746	0.853	0.658	0.795	0.654	0.682	0.794	0.725	0.674							
TS	TS1	TS2	TS3	TS4	TS5	TS6	TS7	TS8	TS9						0.59	0.77	0.95	0.86
Estimate	0.842	0.821	0.612	0.756	0.826	0.742	0.674	0.769	0.721									
PS	PS1	PS2	PS3	PS4	PS5	PS6	PS7	PS8	PS9	PS10					0.54	0.70	0.92	0.76
Estimate	0.842	0.81	0.778	0.8321	0.714	0.682	0.874	0.618	0.586	0.554								
Anx	Anx1	Anx2	Anx3	Anx4	Anx5	Anx6	Anx7	Anx8	Anx9	Anx10	Anx11	Anx12	Anx13		0.51	0.71	0.93	0.79
Estimate	0.665	0.632	0.74	0.753	0.689	0.74	0.669	0.771	0.738	0.762	0.734	0.711	0.574					

SA = Social adjustment, SE = Self-efficacy, TS = Teachers’ support, PS = Peer support, Anx = Anxiety, AVE = average variance extracted, C.R. = composite reliability.

**Table 3:** Effect of gender on social adjustment of students.

Variable	Gender	M (SD)	t-Value	*p*-Value	Cohen’s d	Effect Size
Social adjustment	Boys	41.33 (10.39)	6.983	<0.001	0.97	0.44
	Girls	50.11 (7.45)				

**Table 4:** Regression analysis for the effect of self-efficacy, teachers’ support, peers’ support, and anxiety on social adjustment.

Predictors	*β*	*t*	*p* (<0.05)	F	R^2^	adjR^2^
**SE**	0.796	14.862	<0.05	220.874	0.424	0.426
**TS**	0.729	13.057	<0.05	170.489	0.364	0.362
**PS**	0.753	12.864	<0.05	165.484	0.356	0.358
**AN**	−0.402	−8.047	<0.05	65.753	0.178	0.176

## Data Availability

The data presented in this study are available on request from the corresponding author. The data are not publicly available due to ethical concerns.
